# Supplementary material for: State aggregation for fast likelihood computations in molecular evolution
Source: Bioinformatics. 2016 Oct 2;33(3):354–62. doi: 10.1093/bioinformatics/btw632 (PMC5408795; doi:10.1093/bioinformatics/btw632)
Supplement: Supplementary Data [file btw632_supp.pdf]

Supplementary information

State aggregation for fast likelihood computations in molecular evolution

Iakov I. Davydov, Marc Robinson-Rechavi, Nicolas Salamin*

Department of Ecology and Evolution, Biophore, University of Lausanne, 1015 Lausanne, Switzerland
Swiss Institute of Bioinformatics, Genopode, Quartier Sorge, 1015 Lausanne, Switzerland

*Corresponding author, nicolas.salamin@unil.ch

Dataset	Sequence length	Number of sequences	ω_0	κ	Codon frequencies	Tree length	Number of sequences
`wvar`	300	18	$\sim Beta(2,5)$	2	1/61	4	200
`kvar`	300	18	0.3	$\sim Unif(1/2, 10)$	1/61	4	200
`alen`	100–5000	18	0.3	2	1/61	4	200
`nseq`	300	8–50	0.3	2	1/61	4	200
`tlen`	300	18	0.3	2	1/61	10^p<br>$p \sim Unif(-4, 4)$	200
`cfreq`	300	18	0.3	2	$\sim Direchlet(\alpha)$<br>$\alpha \sim 10^{Unif(-1/2,1)}$	4	200

Table S1: List of simulated datasets for M0 model.

A

Parameter	Distribution
κ	$1 + Exponential(1)$
ω_0	$Beta(2,5)$
ω_2	$1 + Gamma(10,2)$, ($= 1$ for H0)
$p_0 + p_1$	$Beta(10,1)$
$\frac{p_0}{p_0+p_1}$	$Beta(10,1)$
Tree length	$Gamma(2,2)$
Number of codons	$Unif(100,1000)$
Number of sequences	$Unif(8,30)$

B

Parameter	Distribution
α (*Beta* distribution parameter, negative selection)	$Gamma(5,1)$
β (*Beta* distribution parameter)	$Gamma(8,1)$
$Mean(\omega_2)$ (mean of the *Gamma* distribution, positive selection)	$1 + Gamma(10,2)$, ($\omega_2 = 1$ for H0)
$Var(\omega_2)$ (variance of the *Gamma* distribution)	$Beta(20,50) \cdot Mean(\omega_2)$
α (shape of the *Gamma* distribution for the site rate variation)	$\frac{1}{2} + Exponential(\frac{1}{4})$

Table S2: Model parameter distribution the simulated datasets A) branch-site model; B) extra parameters for the extended branch-site model.

A		Selection detected (aggregated)	
		−	+
Selection detected	−	79140	5
(normal)	+	7	13

B		Selection detected (aggregated)	
		−	+
Selection detected	−	79054	24
(normal)	+	27	60

Table S3: Statistical performance of FastCodeML on the Primates dataset. Detected selection in normal and aggregated modes of FastCodeML. Numbers in the cells correspond to the number of performed tests. Every non-terminal branch was tested. A) After correction for multiple hypothesis testing, FDR (false discovery rate) cutoff=0.05; B) FDR cutoff=0.4.

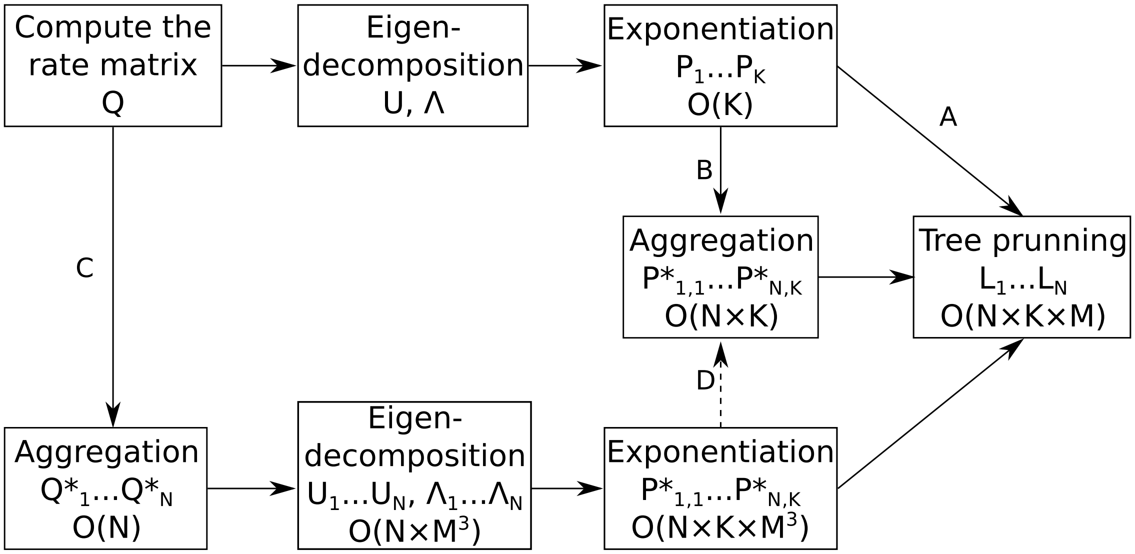

Figure S1: Schematic representation of the tree likelihood computation: A) full likelihood; B) post-exponentiation aggregation; C) pre-exponentiation aggregation; D) pre- and post-exponentiation aggregation. Rough algorithm complexity indicated for steps dependent on alignment length (N), internal nodes count (K) and dimensionality of aggregated Markov chain (M).

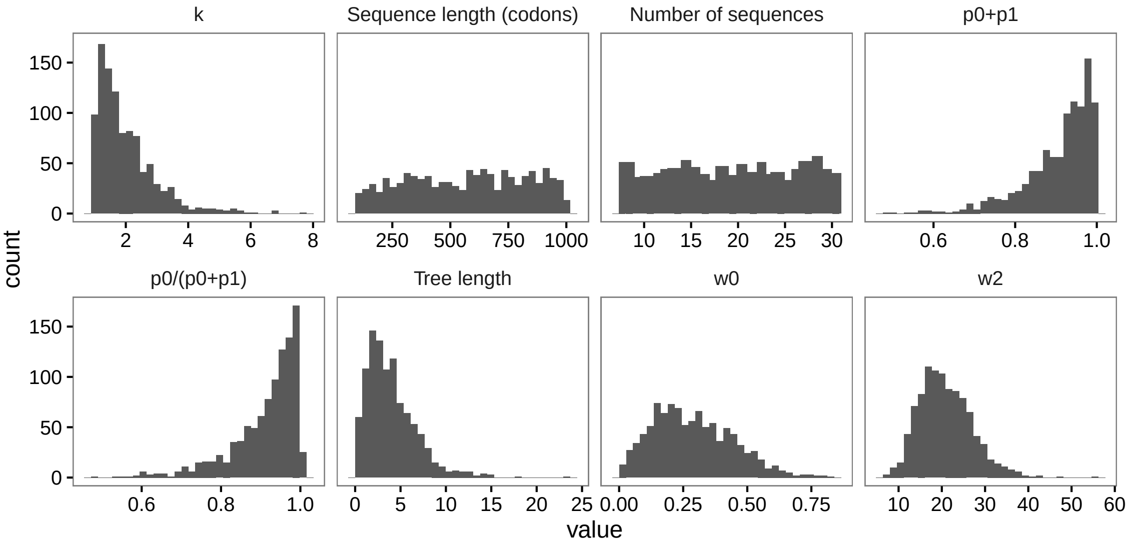

Figure S2: Parameter distribution for branch-site model simulations.

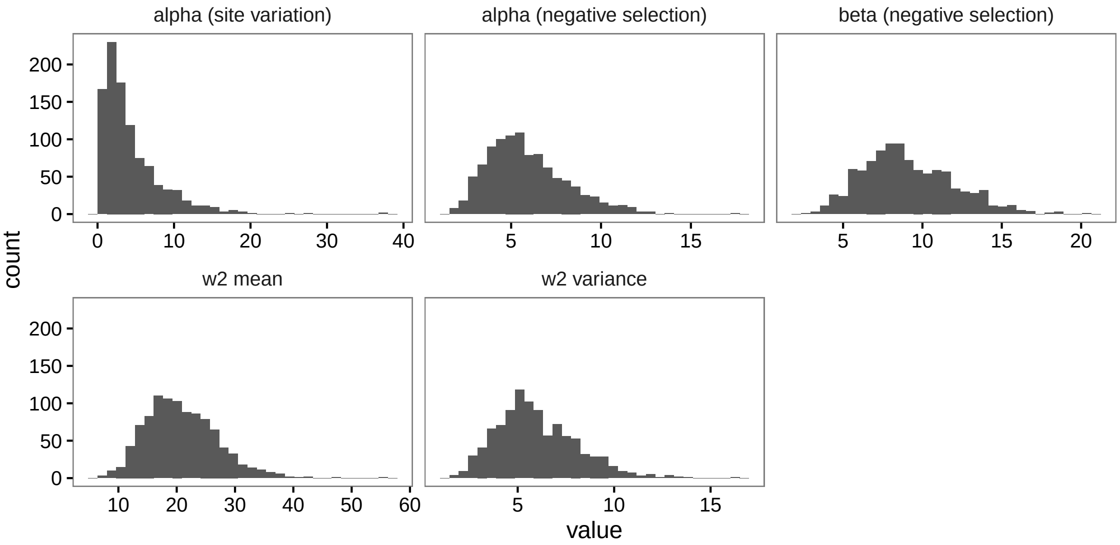

Figure S3: Parameter distribution for extended branch-site model simulations. See text for the parameter descriptions.

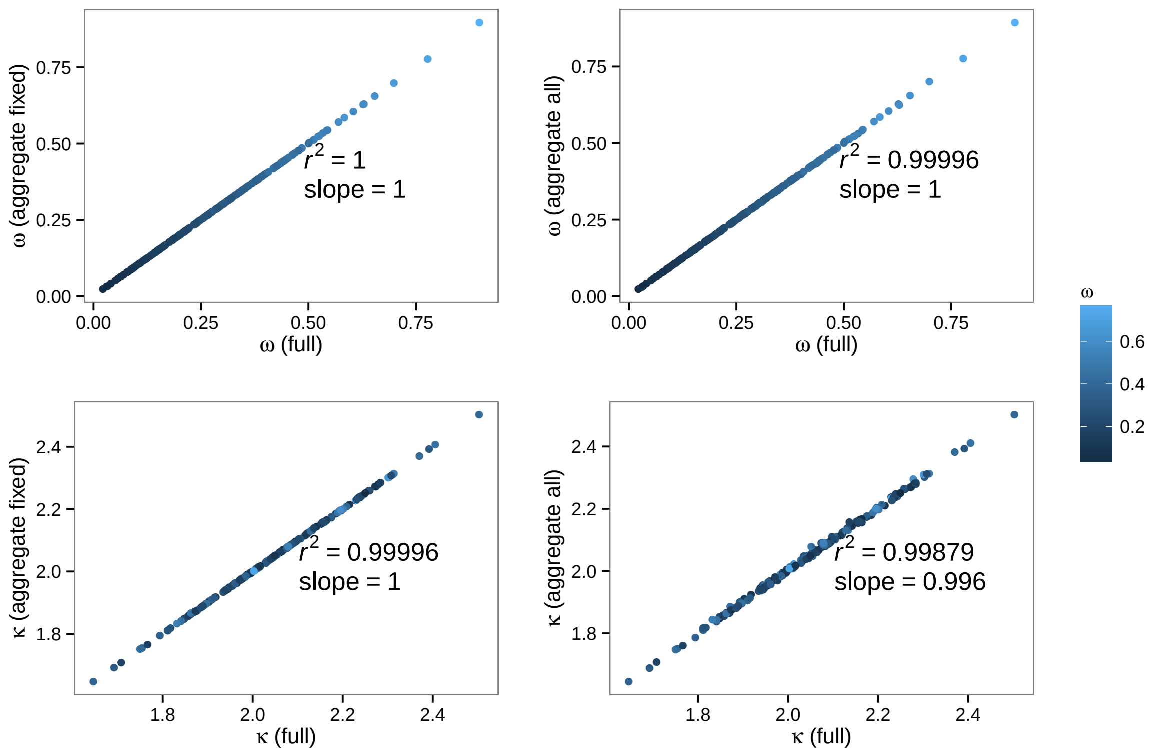

Figure S4: Correlation between estimated ω and κ values in normal and aggregated modes for varying simulated ω value (`wvar` dataset, M0 model).

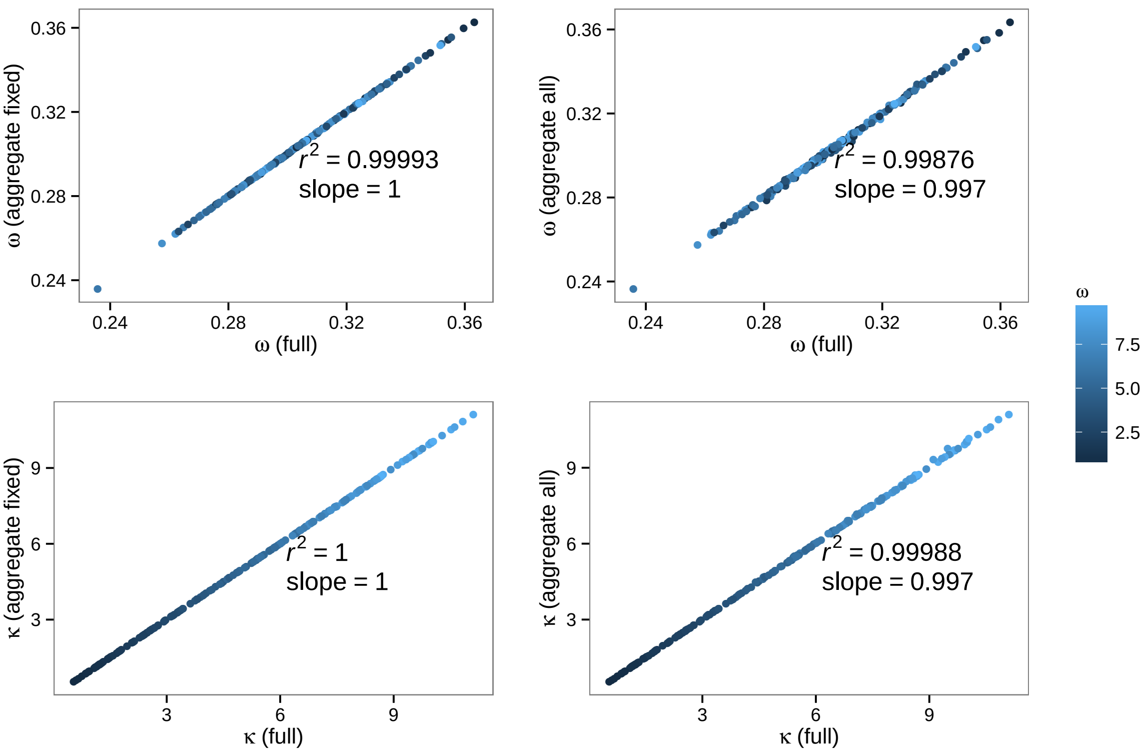

Figure S5: Correlation between estimated ω and κ values in normal and aggregated modes for varying simulated κ value (`kvar` dataset, M0 model).

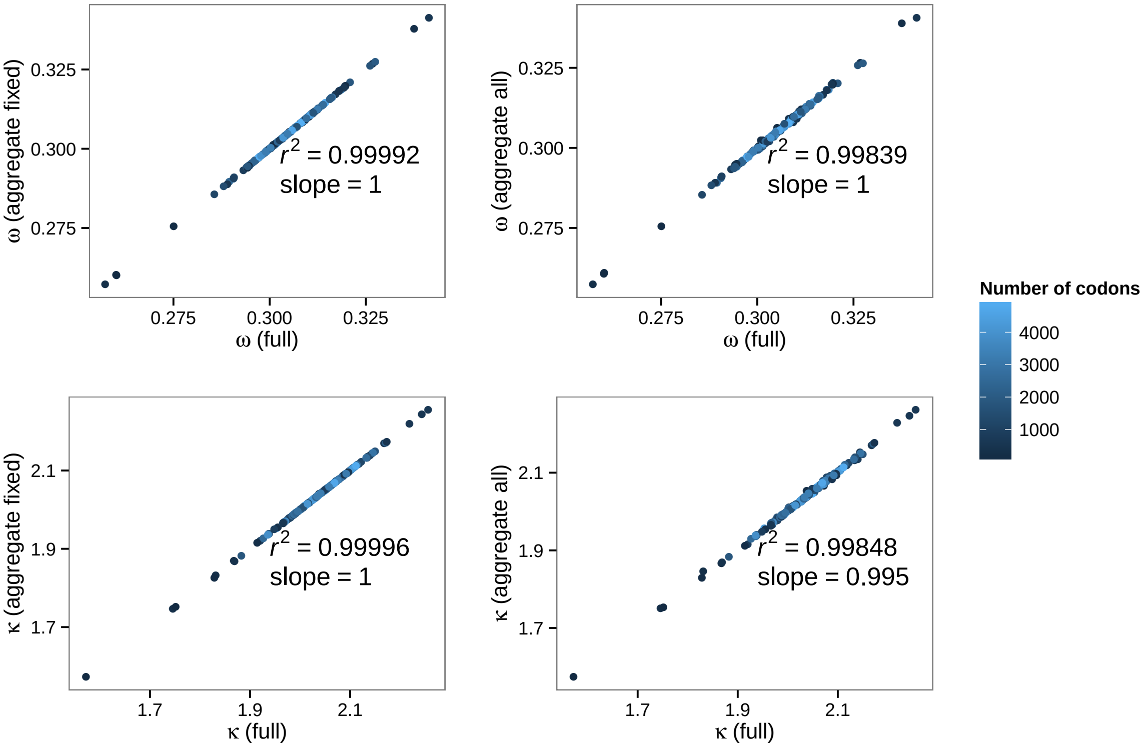

Figure S6: Correlation between estimated ω and κ values in normal and aggregated modes for varying simulated sequences length (`alen` dataset, M0 model).

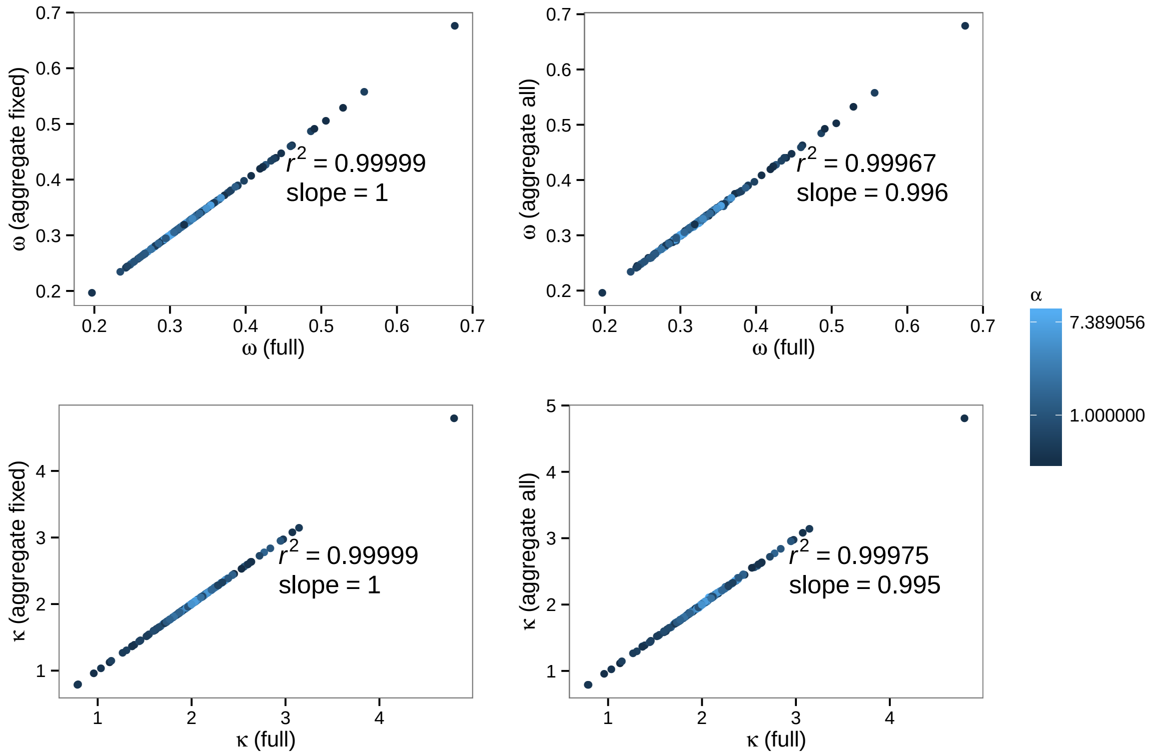

Figure S7: Correlation between estimated ω and κ values in normal and aggregated modes for varying codon frequencies Dirichlet distribution α parameter value (`cfreq` dataset, M0 model).

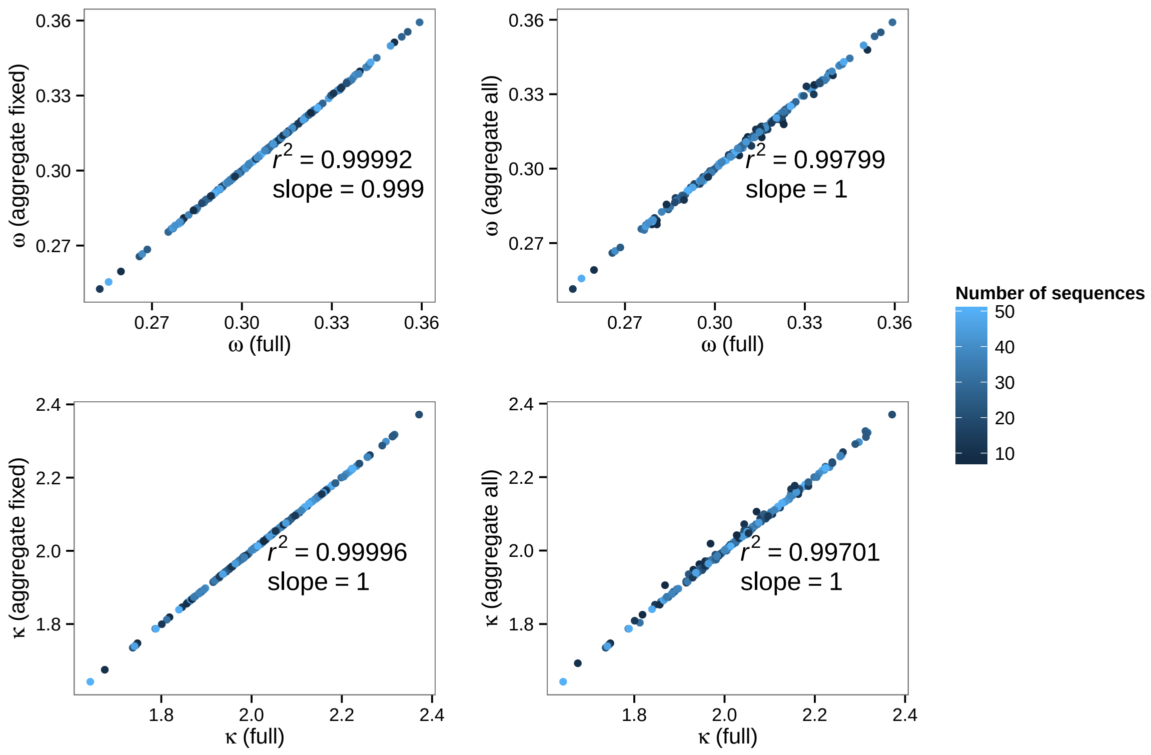

Figure S8: Correlation between estimated ω and κ values in normal and aggregated modes for varying number of simulated sequences (`nseq` dataset, M0 model).

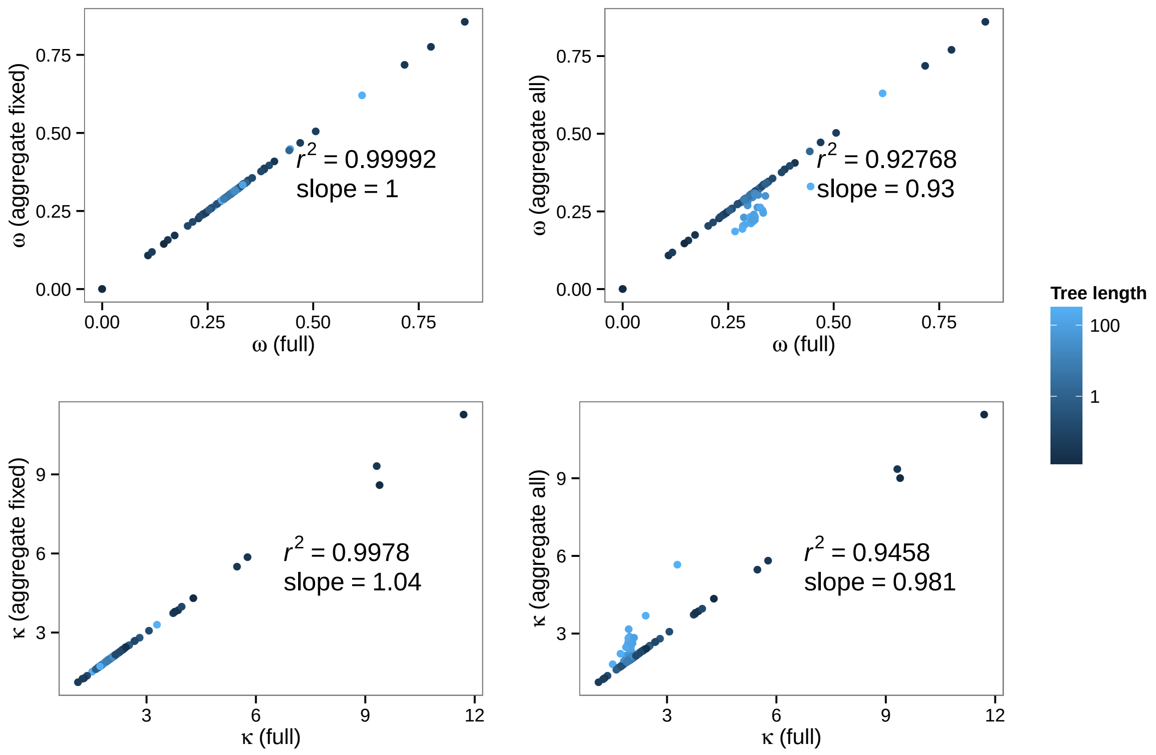

Figure S9: Correlation between estimated ω and κ values in normal and aggregated modes for varying tree length (`tlen` dataset, M0 model). Tree length limited to the range $[0.01; 300]$, see text.

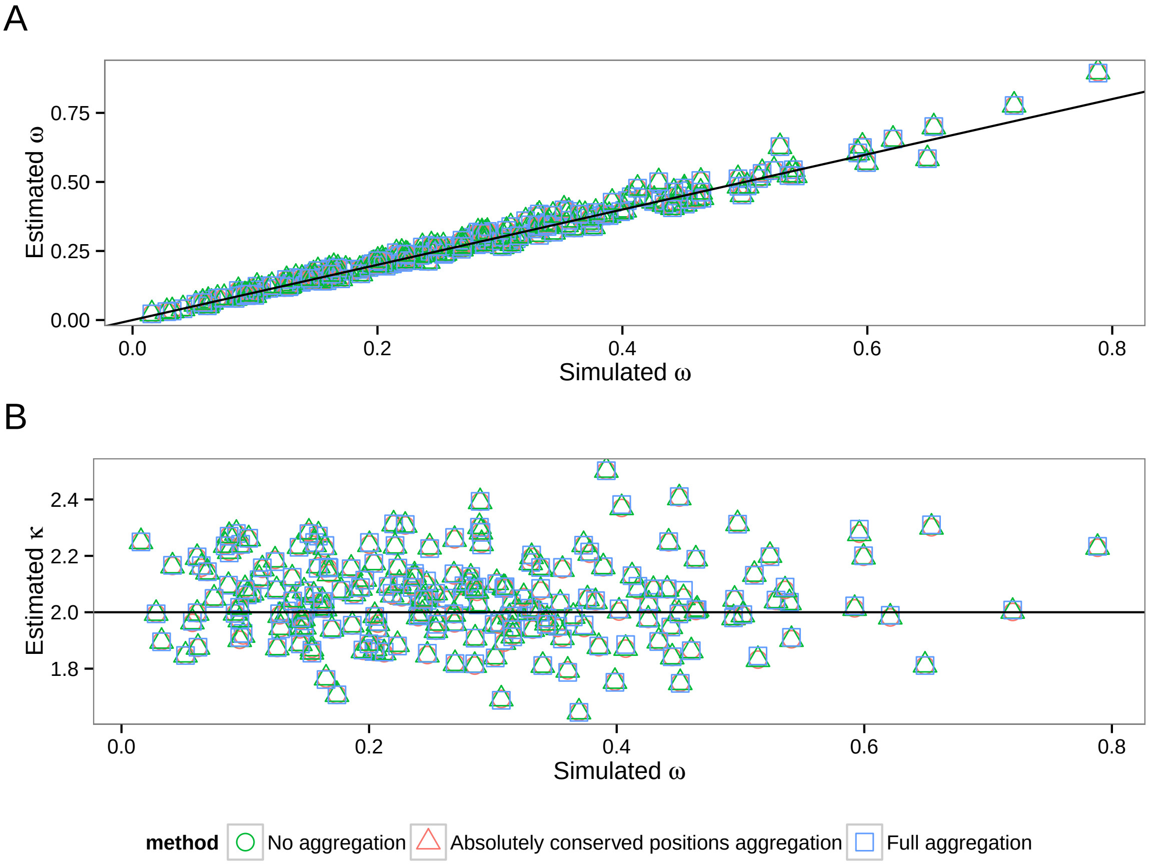

Figure S10: Estimated ω (A) and κ (B) values versus simulated ω value for the `wvar` dataset, M0 model. Lines correspond to the simulation parameter values.

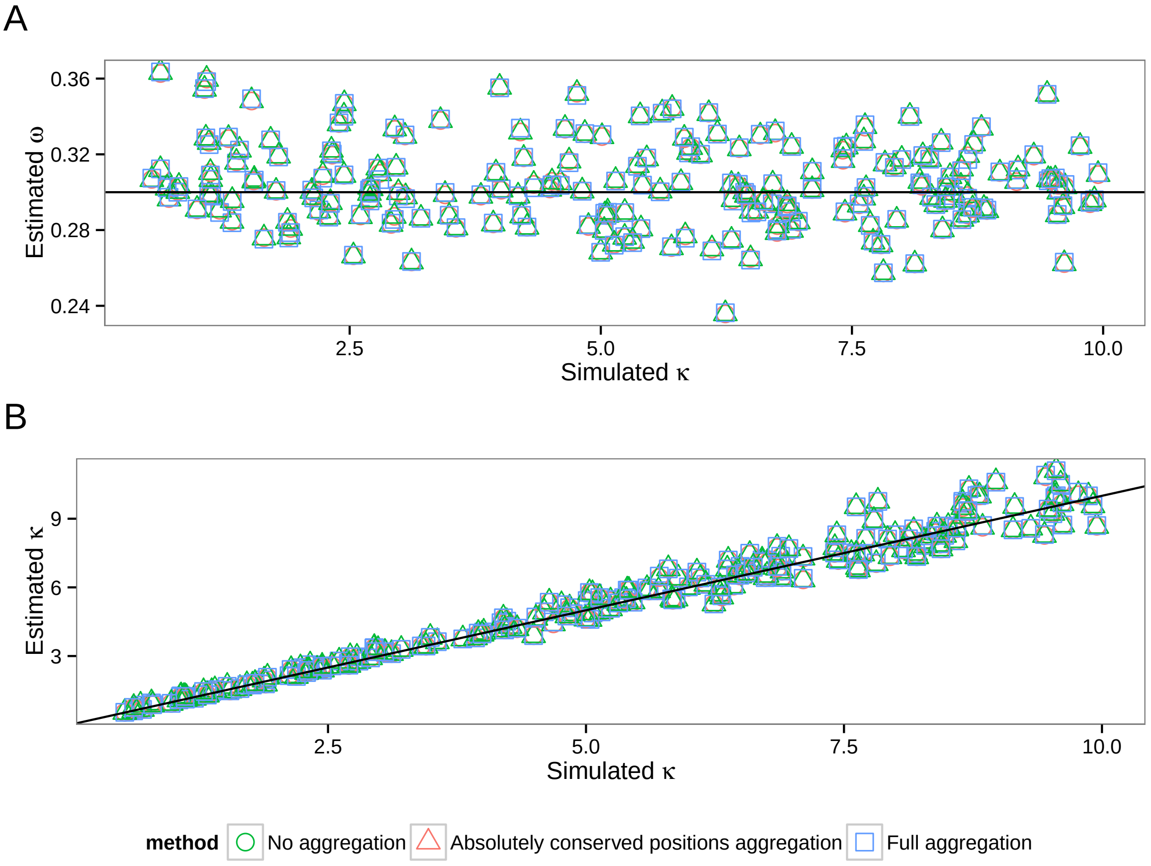

Figure S11: Estimated ω (A) and κ (B) values versus simulated κ value for the `kvar` dataset, M0 model. Lines correspond to the simulation parameter values.

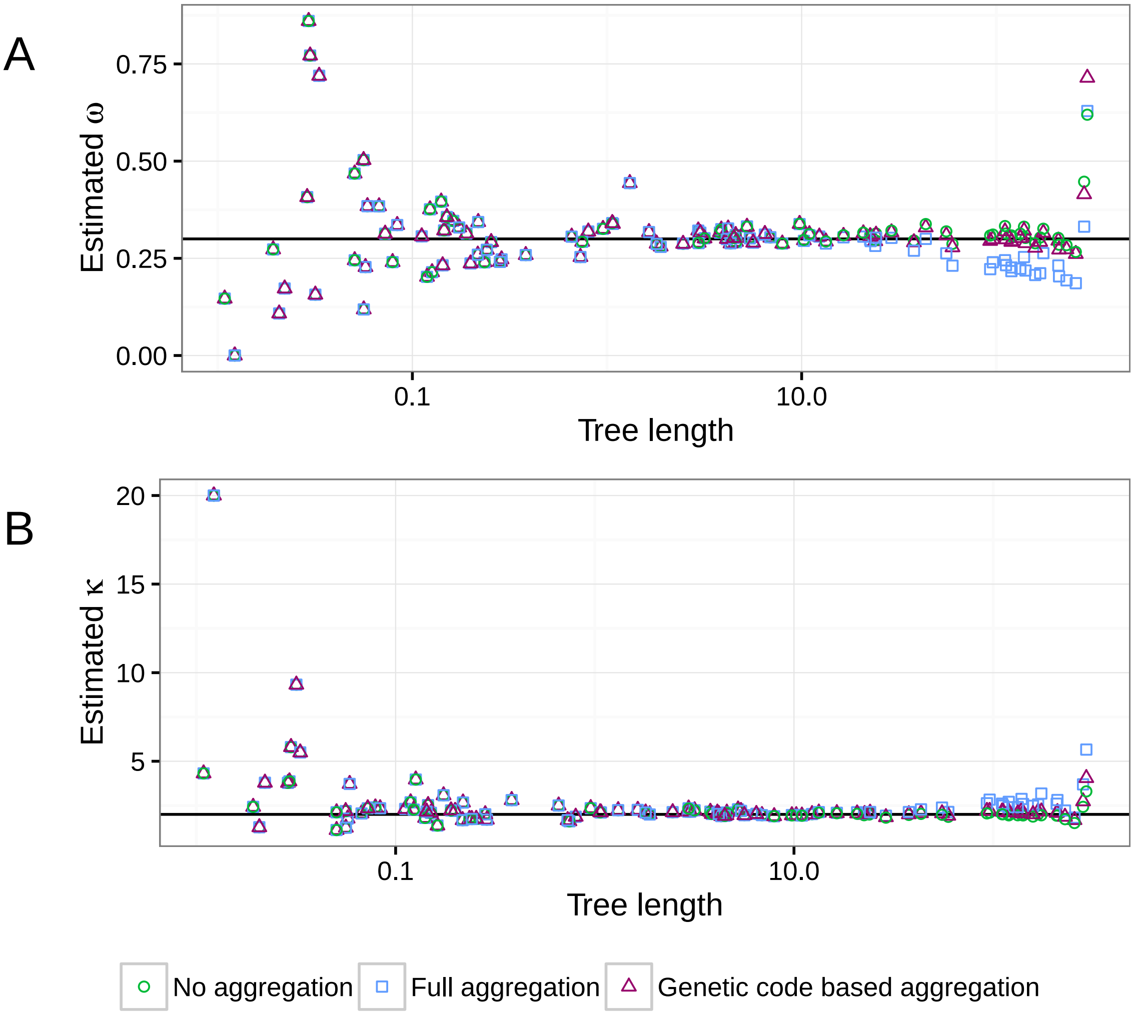

Figure S12: Estimated ω (A) and κ (B) values versus simulated tree length for the `tlen` dataset, M0 model. Lines correspond to the simulation parameter values. Tree length limited to the range $[0.01; 300]$, see text. Optimization with a variable number of iterations using the Broyden–Fletcher–Goldfarb–Shanno algorithm variant (L-BFGS-B).

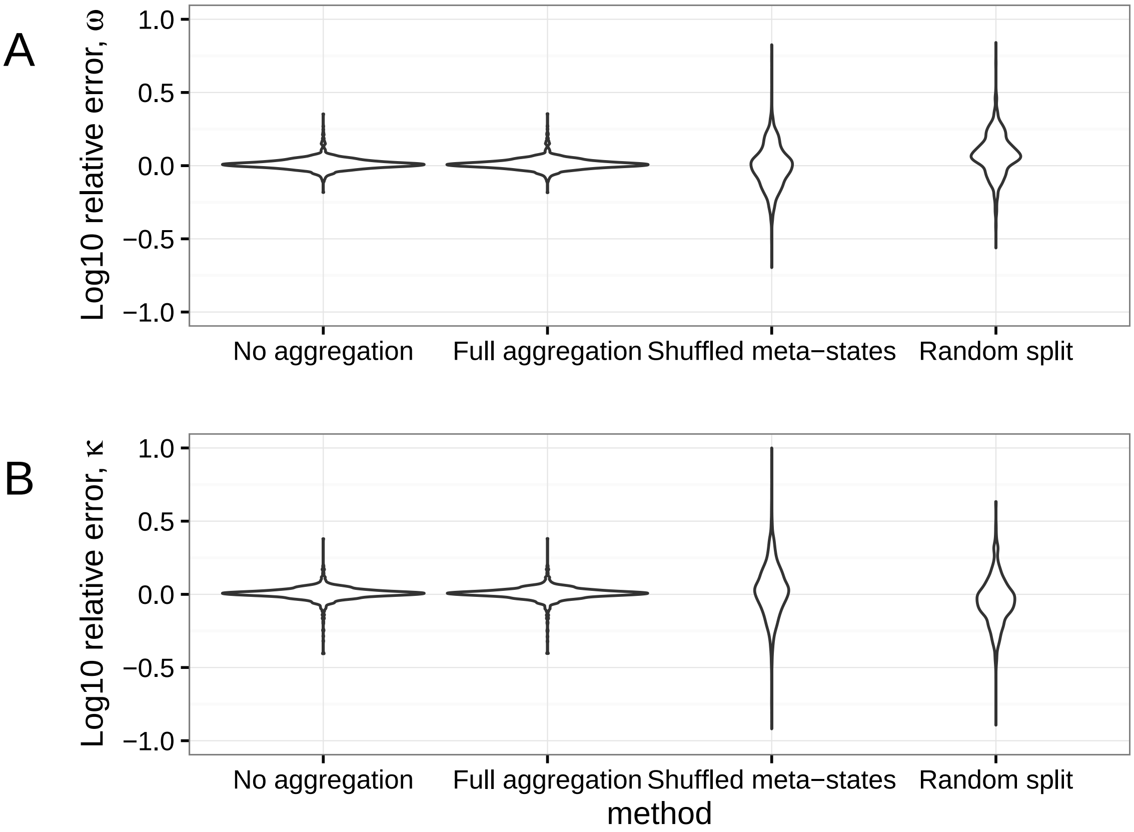

Figure S13: Relative error (maximum likelihood estimate divided by the true value) of ω (A) and κ (B) estimation using various aggregation strategies. All the M0 datasets except for `tlen` were used. Optimization was performed using the L-BFGS-B algorithm.

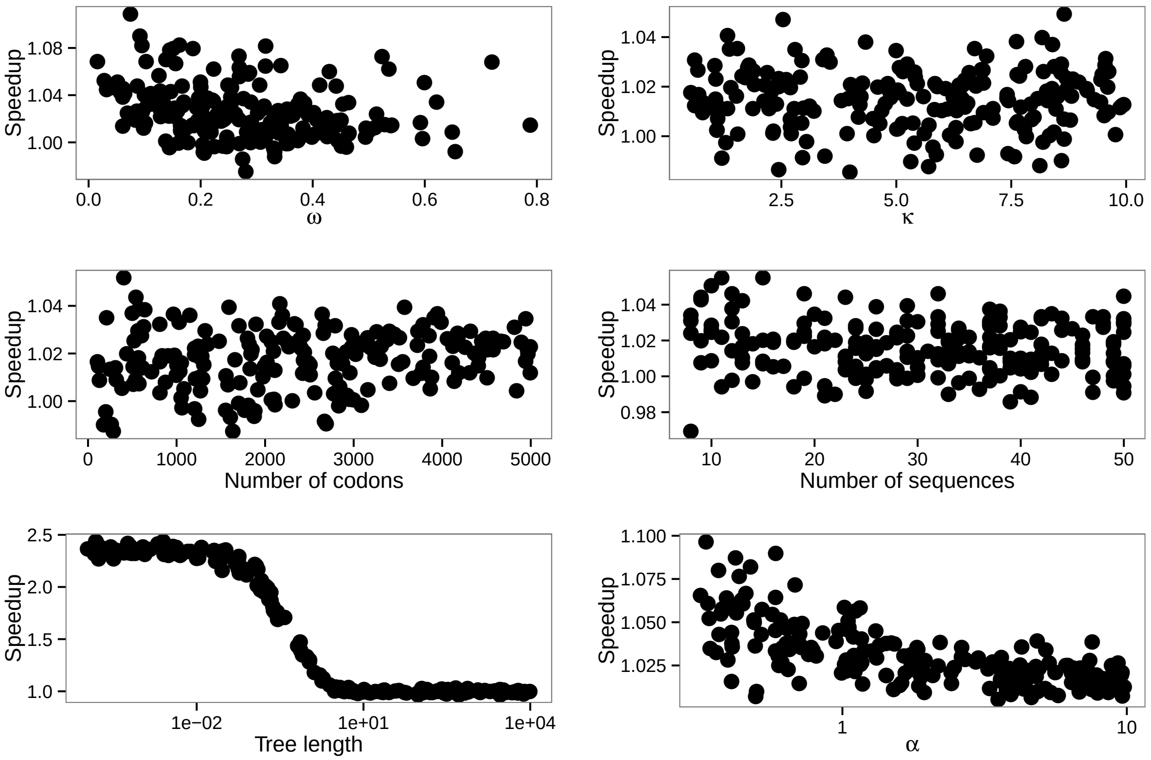

Figure S14: Speedup for fixed-positions only aggregation, M0 model.

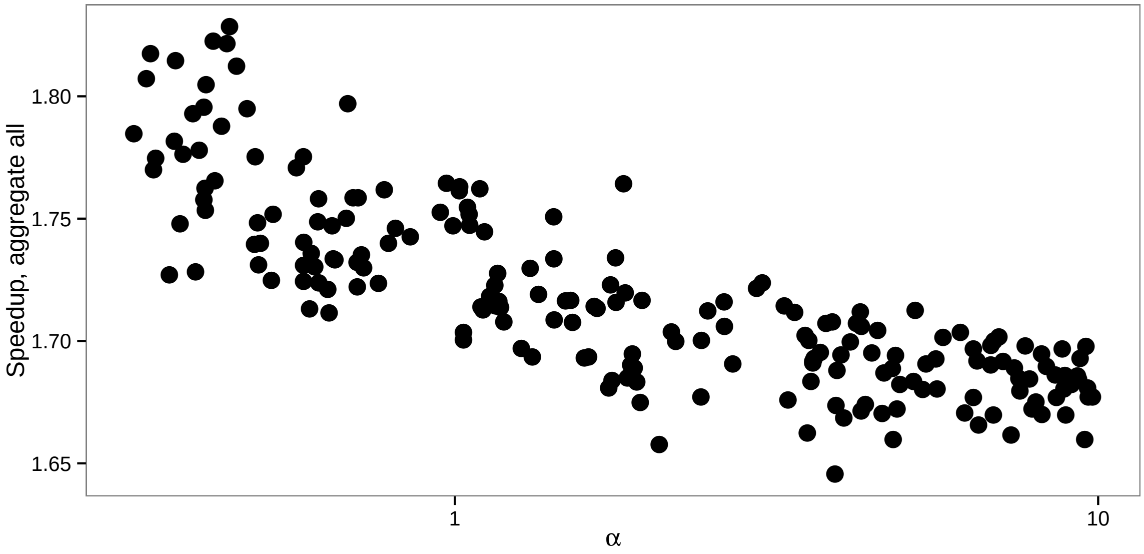

Figure S15: Speedup versus the α parameter of the codon frequencies Dirichlet distribution, M0 model, `cfreq` dataset.

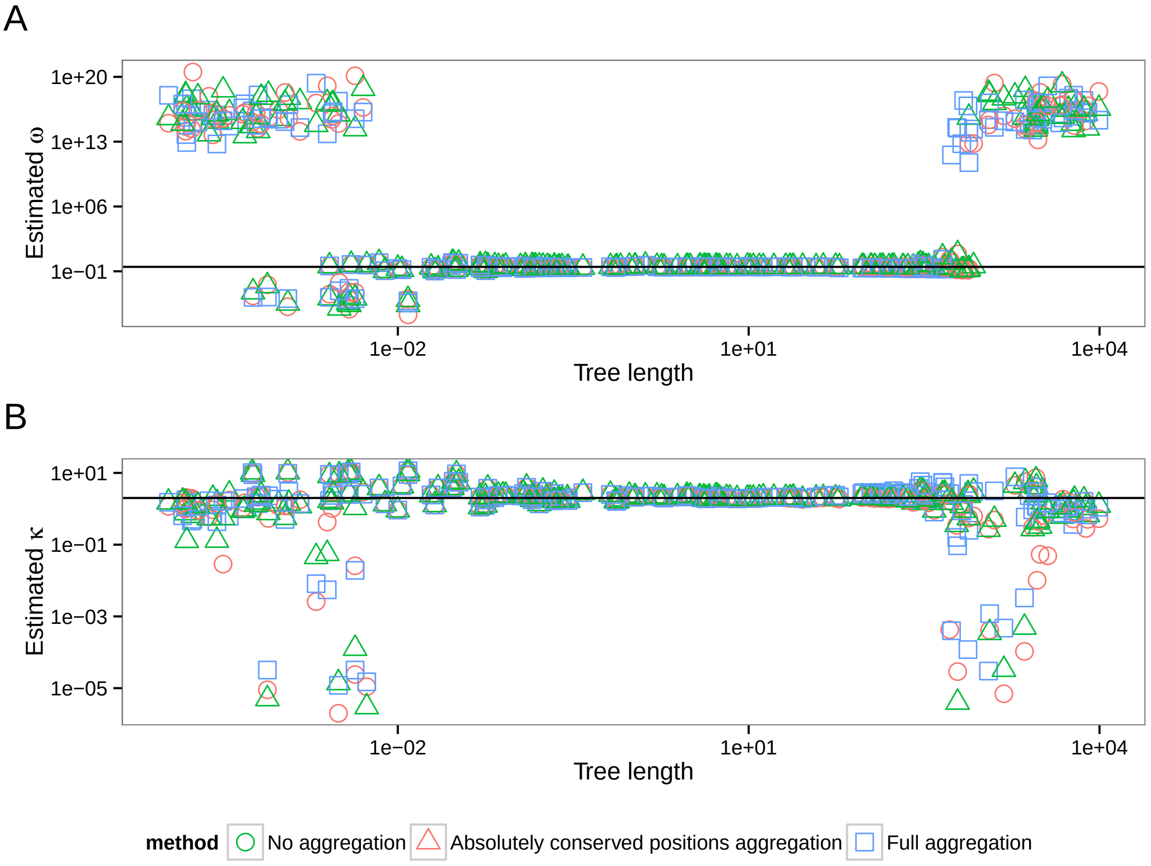

Figure S16: Estimated ω (A) and κ (B) values versus simulated tree length for the `tlen` dataset, M0 model. Lines correspond to the simulation parameter values.

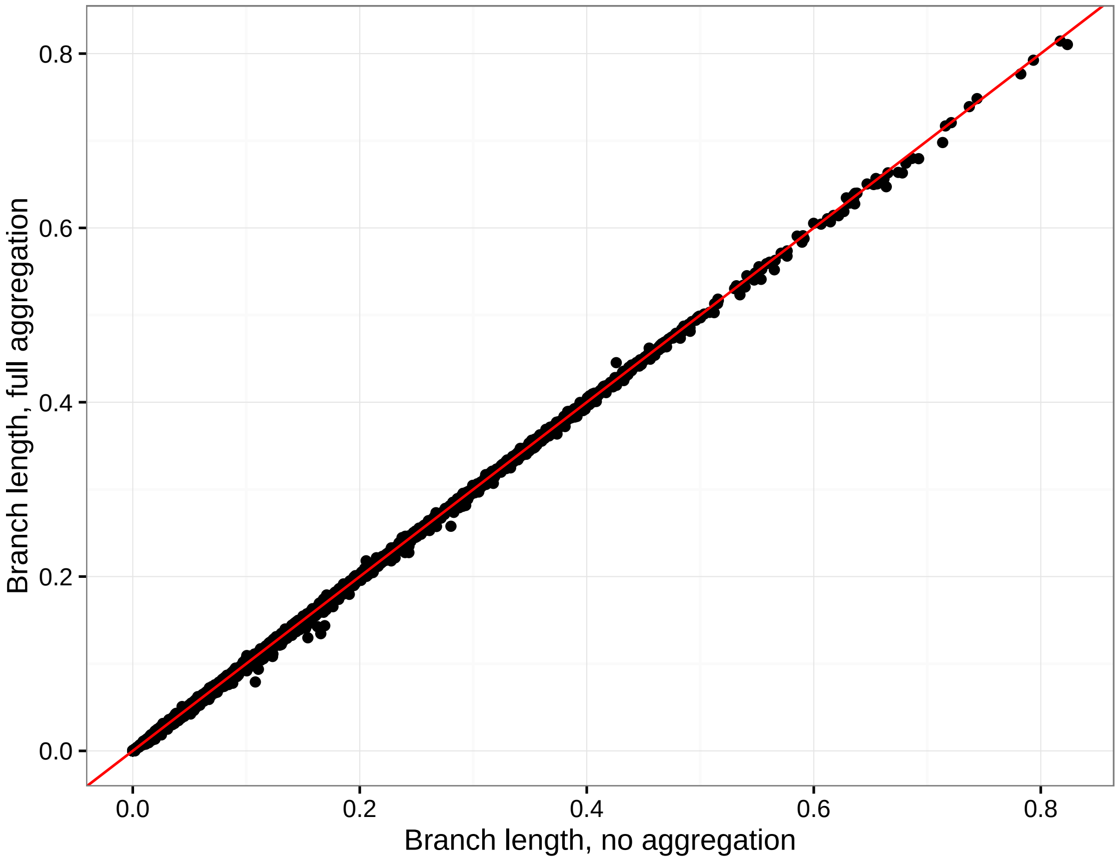

Figure S17: Branch lengths estimated with and without aggregation, M0 model. Each data point represents an individual branch from a single tree. This plot includes all the M0 datasets except for the varying tree length dataset (`tlen`). The red line indicates equal values. Optimization performed using the L-BFGS-B algorithm.

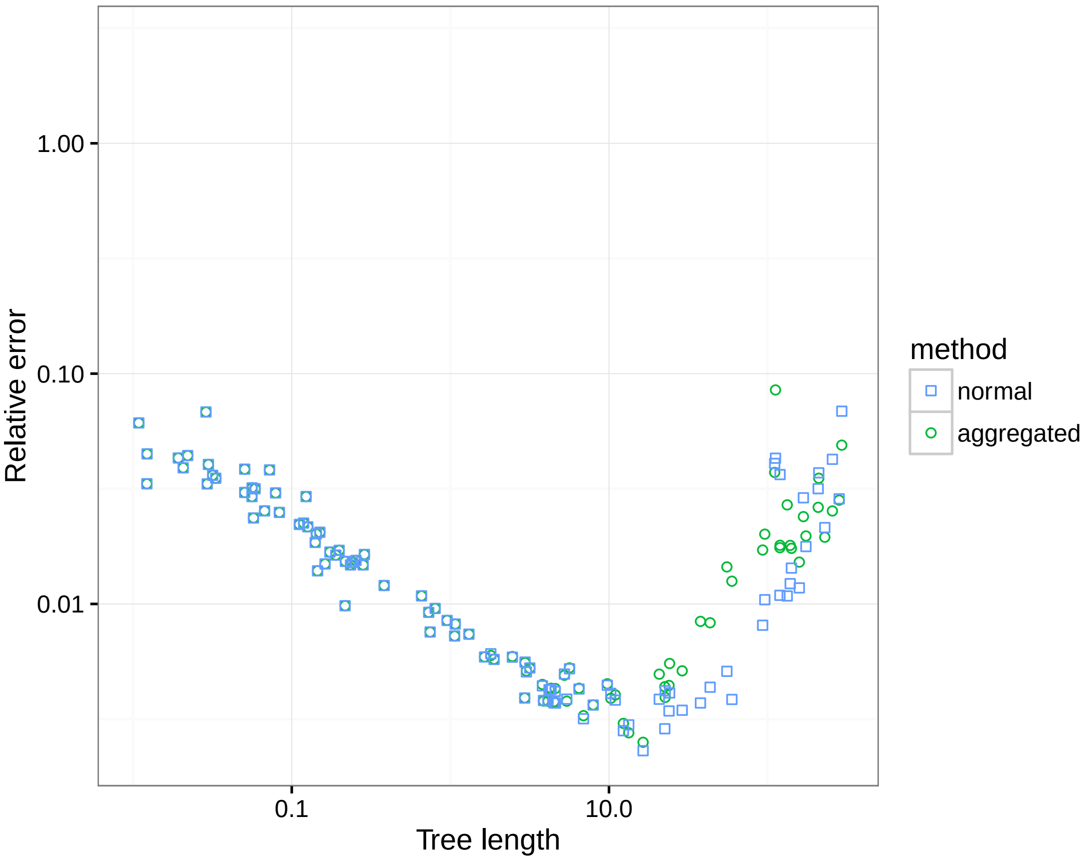

Figure S18: Branch length estimation error versus total tree length for the `tlen` dataset, M0 model. Both axes are log-scale. Relative error (E) for a given tree is defined as $E = \frac{\sum |t^i_{estimated} - t^i_{true}|}{N} / \sum t^i_{true}$, where $t^i_{estimated}$ is an estimated length for branch i, t^i_{true} is a true length and N is a number of branches in a tree. For tree lengths below 10 symbols are overlapping demonstrating almost perfect match. Optimization performed using the L-BFGS-B algorithm. Tree length limited to the range $[0.01; 300]$, see text.

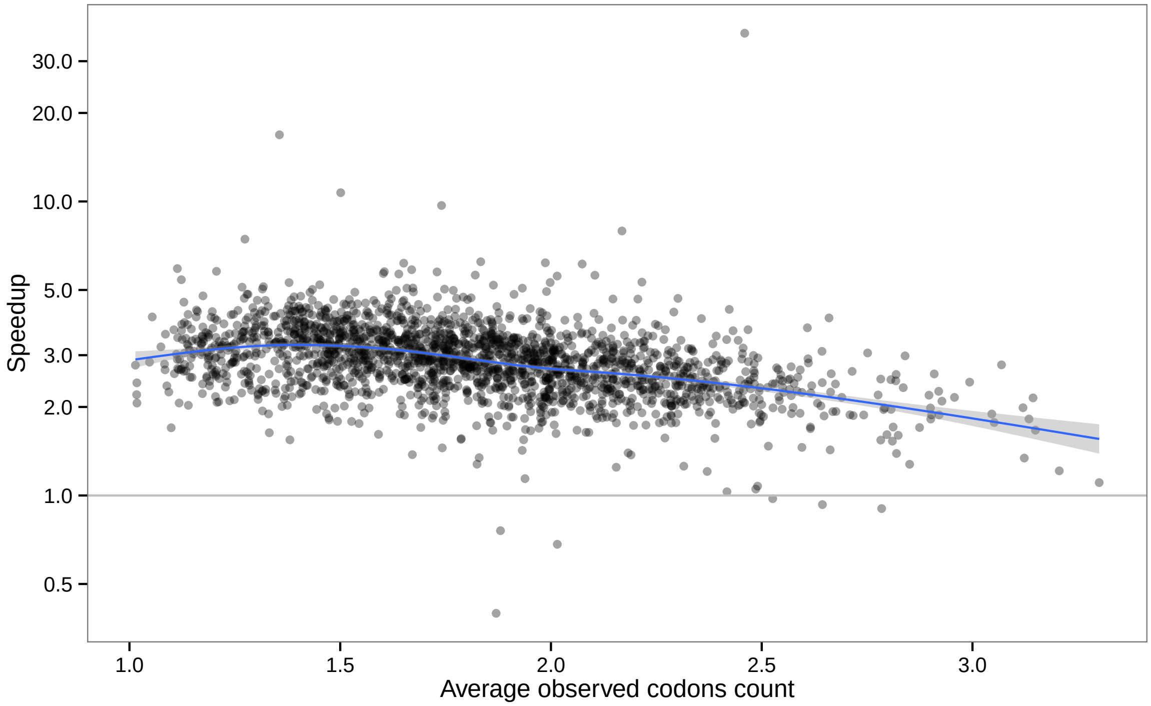

Figure S19: Speedup versus average codon count for the branch-site model. Each point represents one simulated alignment.

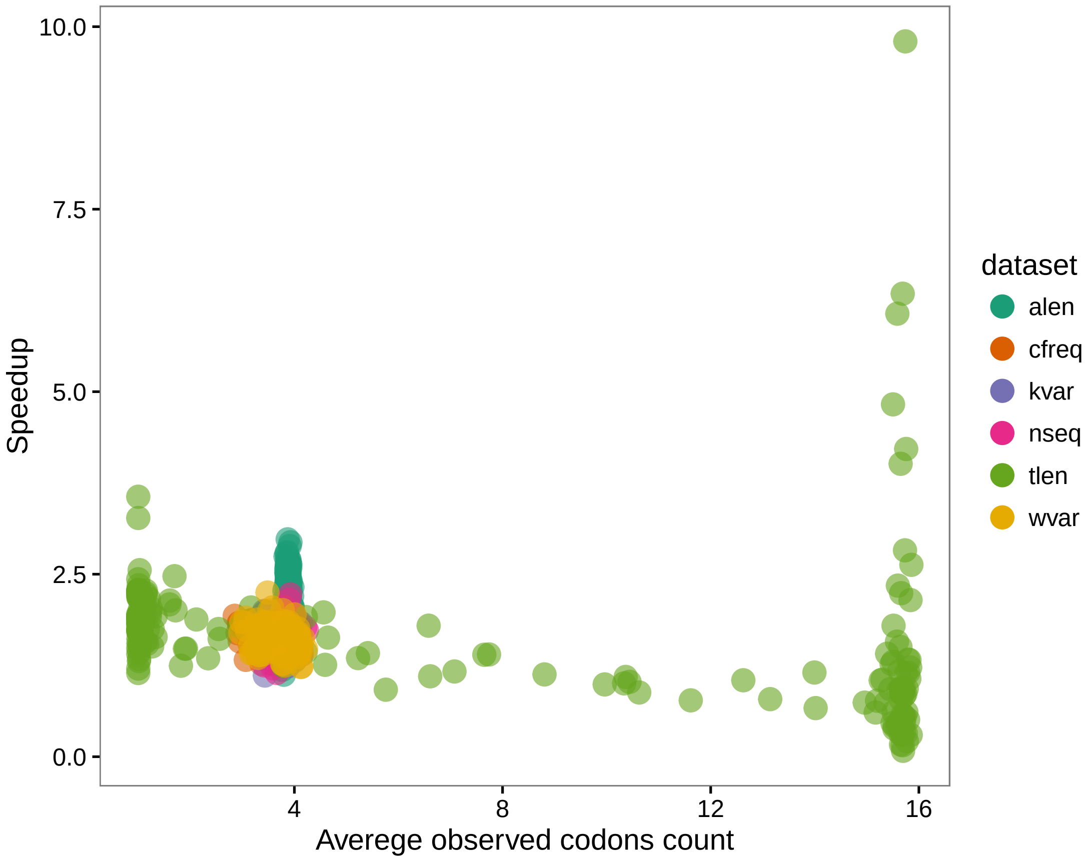

Figure S20: Speedup versus average codon count for M0 model. Optimization performed using the L-BFGS-B algorithm. Branch lengths, ω and κ were optimized.

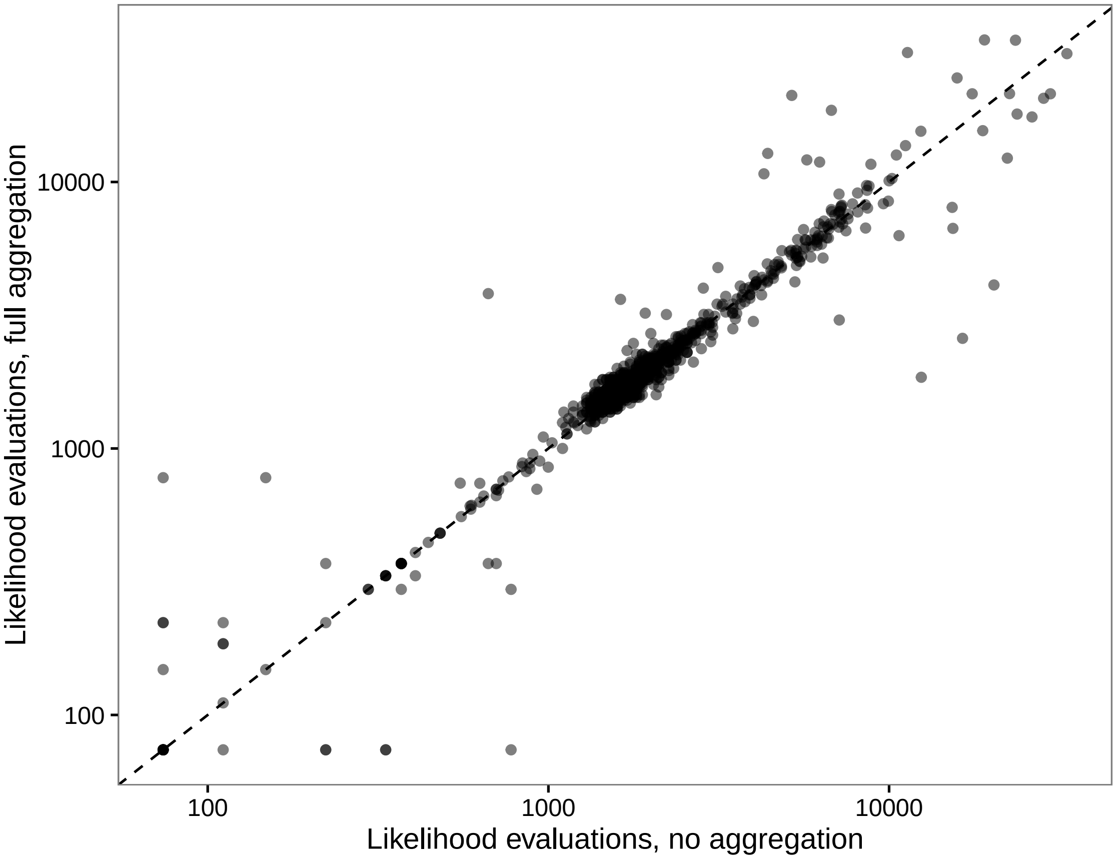

Figure S21: Number of Likelihood evaluations for M0 model with and without state aggregation. Optimization performed using the L-BFGS-B algorithm. Branch lengths, ω and κ were optimized. Both axes are log-scale. Dashed line indicates the ideal match.

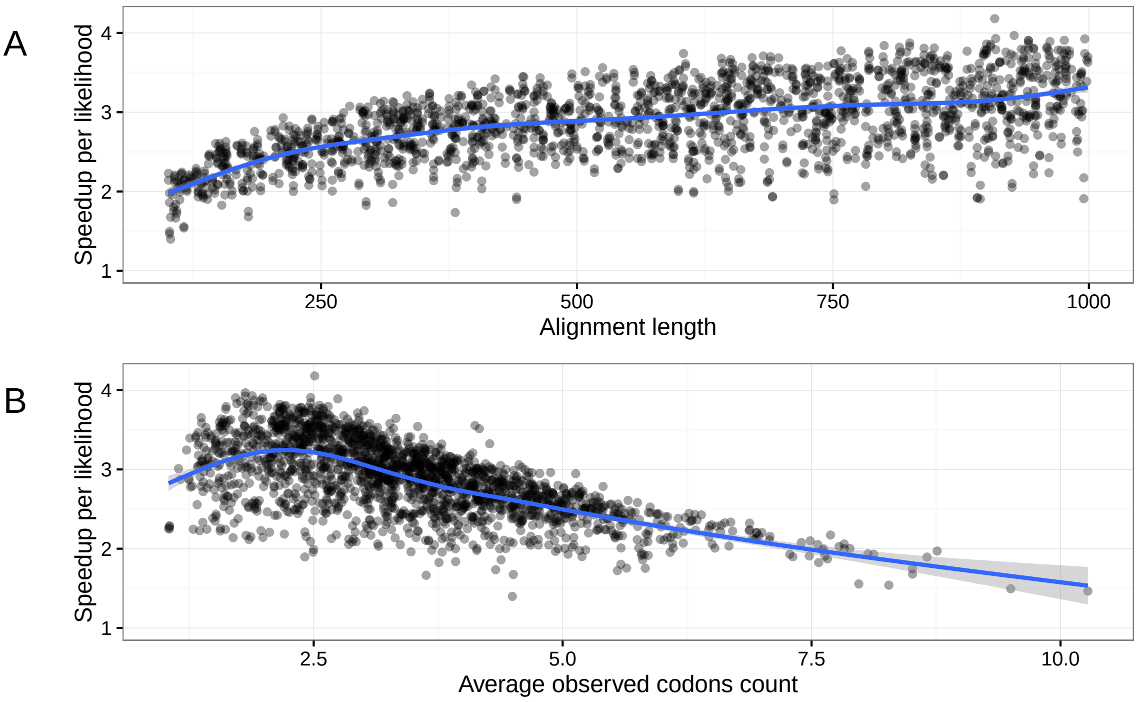

Figure S22: Average speedup per likelihood computation versus A) alignment length and B) average codon count for the branch-site model. Each point represents one simulated alignment.

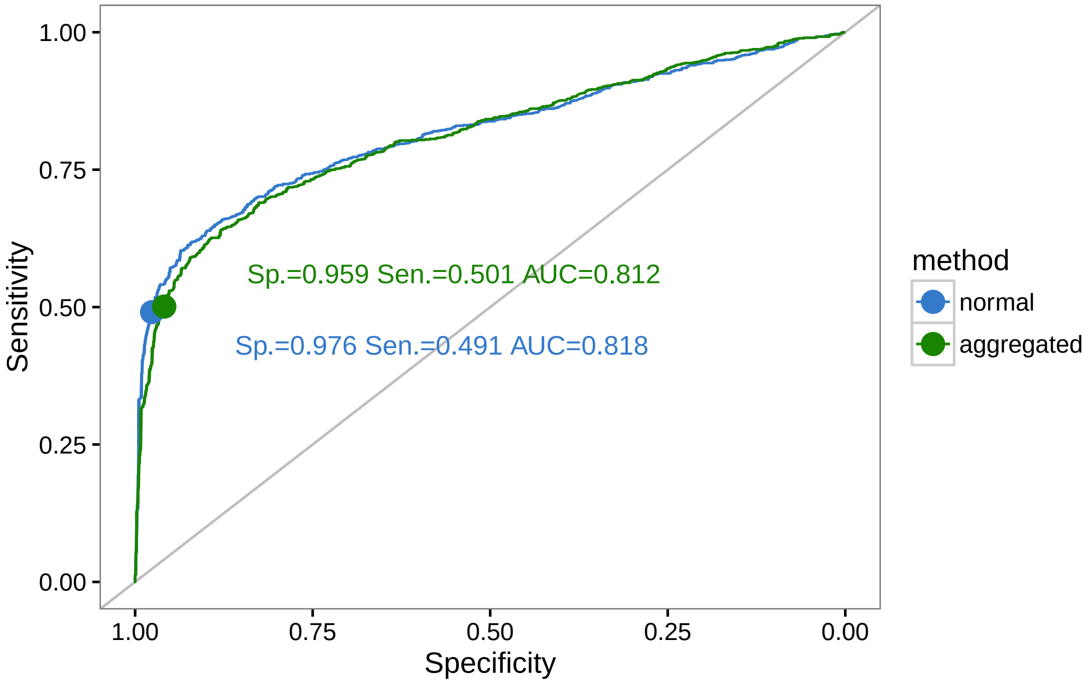

Figure S23: ROC curves for FastCodeML in full likelihood and aggregated likelihood modes for the extended branch-site model simulations. Specificity, sensitivity and AUC indicated.

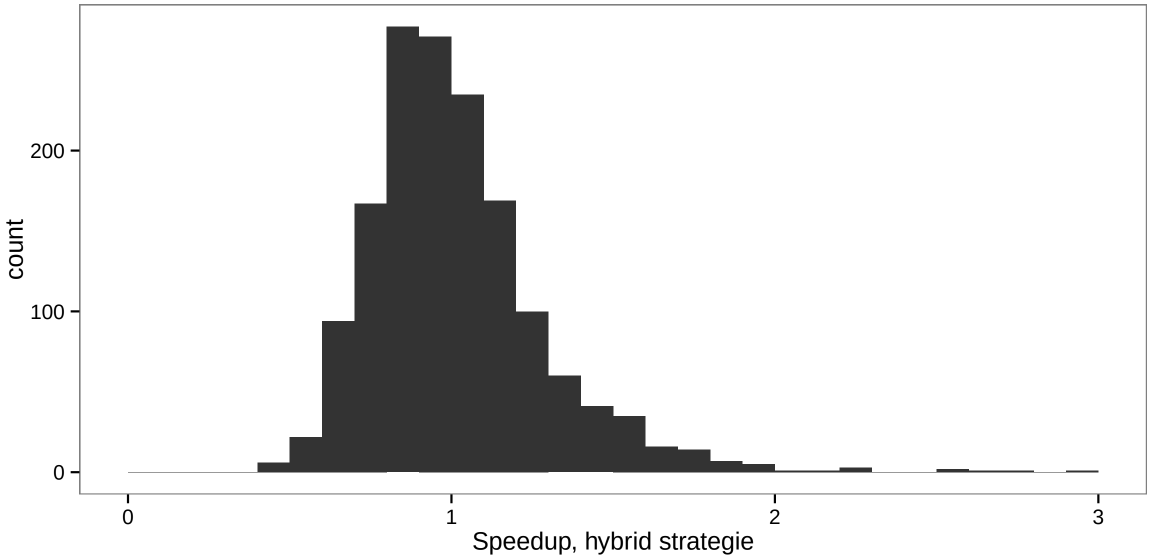

Figure S24: Speedup of hybrid strategy. Maximum likelihood estimation in aggregated mode is followed by full mode likelihood maximization (branch-site model).
